# Squamous Cell Carcinoma in Chronic Cutaneous Leishmaniasis: Coincidence or Malignant Transformation

**DOI:** 10.7759/cureus.84720

**Published:** 2025-05-24

**Authors:** Oumaima Bouraqqadi, Sara Elloudi, Meryem Zaryouhi, Laila Chbani, Fatima Zahra Mernissi

**Affiliations:** 1 Dermatology Department, Hassan II University Hospital, Fez, MAR; 2 Pathology Laboratory, Hassan II University Hospital, Fez, MAR

**Keywords:** cutaneous leishmaniasis, cutaneous malignancy, cutaneous squamous cell carcinoma (scc), old world leishmaniasis, squamous cells carcinoma

## Abstract

We report the case of a 69-year-old man who developed squamous cell carcinoma (SCC) at the site of a chronic cutaneous leishmaniasis (CL) lesion on the dorsum of his left hand. The lesion had persisted for over three years despite multiple local and systemic treatments. Histopathological examination confirmed the diagnosis of well-differentiated SCC. The patient was lost to follow-up for a year and returned with locally advanced disease, ultimately requiring hand amputation. This case underscores the potential for malignant transformation in chronic leishmaniasis lesions and highlights the need for timely biopsy and long-term monitoring of atypical or non-healing ulcers in endemic regions.

## Introduction

Squamous cell carcinoma (SCC), the second most common non-melanoma skin cancer, can be highly aggressive if left untreated. While ultraviolet (UV) radiation is the primary cause, other risk factors include chemical carcinogens, ionizing radiation, genetic predisposition, and chronic wounds [1]. Chronic ulcerations, whether from burns, fistulas, or infections, are well-established precursors to SCC. One such infectious etiology is cutaneous leishmaniasis (CL), a parasitic skin disease endemic to several regions, including parts of Morocco [2].

Although rare, malignant transformation of long-standing CL lesions into SCC has been reported, posing both a diagnostic and therapeutic challenge. Differentiating between chronic infection and neoplastic change can be particularly difficult in endemic areas where CL is common and resources may be limited.

Here, we present a case of SCC arising in a chronic, non-healing CL lesion in an elderly man from an endemic region in Morocco. This case underscores the importance of maintaining a high index of suspicion for malignant transformation in persistent infectious dermatoses and highlights the need for vigilant long-term follow-up of chronic cutaneous ulcers in leishmaniasis-endemic areas.

## Case presentation

A 69-year-old man residing in Taza, a region in northeastern Morocco endemic for Leishmania tropica [2], first noticed an itchy red papule on the dorsum of his left hand, which appeared shortly after what he described as an insect bite in 2020, during the SARS-CoV-2 pandemic. Over several weeks, the lesion enlarged, crusted, and became painful. He applied several traditional plant-based remedies, but the lesion progressed to a large ulcer with surrounding erythema.

He eventually sought care at a rural healthcare facility, where a direct smear confirmed the presence of Leishmania amastigotes. Despite several local and oral treatments, the ulcer persisted. An eight-week course of intralesional meglumine antimoniate led to only partial improvement, and repeat smear testing remained positive, prompting referral to our dermatology department for further management.

**Figure 1:**
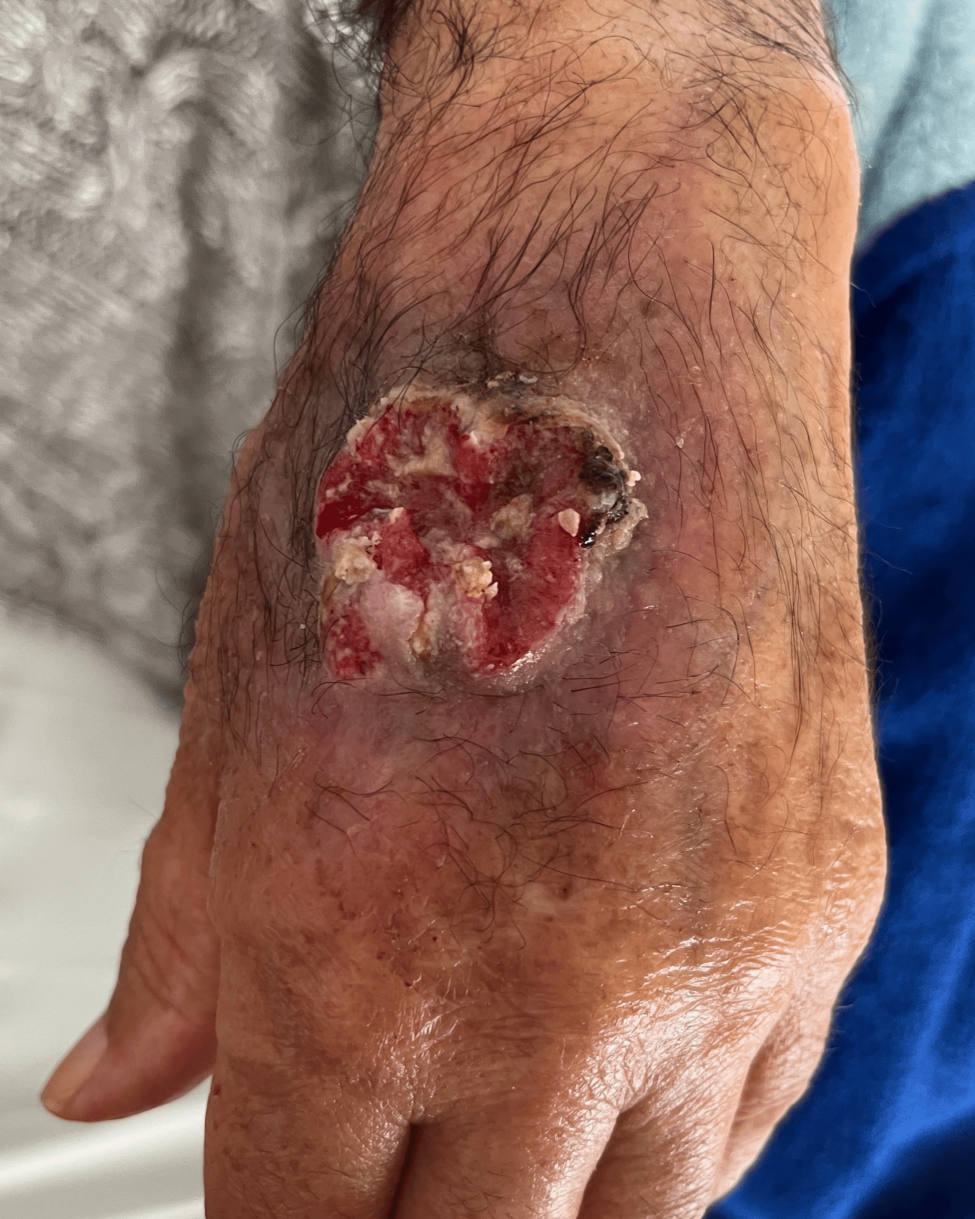
Clinical appearance of the lesion on the dorsum of the left hand at initial presentation, following local wound care. A well-demarcated, approximately 3 cm erythematous ulcer with a fibrino-purulent base, overlying crusts, and peripheral scaling is noted immediately prior to the initiation of systemic meglumine antimoniate therapy.

On clinical examination, the patient presented with a solitary, approximately 3 cm diameter erythematous plaque with a crateriform ulcer, featuring a fibrino-purulent surface, crusting, and scaling on the dorsum of his left hand (Figure 1). The lesion was roughly round in shape. A dermoscopic examination showed no specific patterns. There were no palpable axillary lymph nodes.

**Figure 2:**
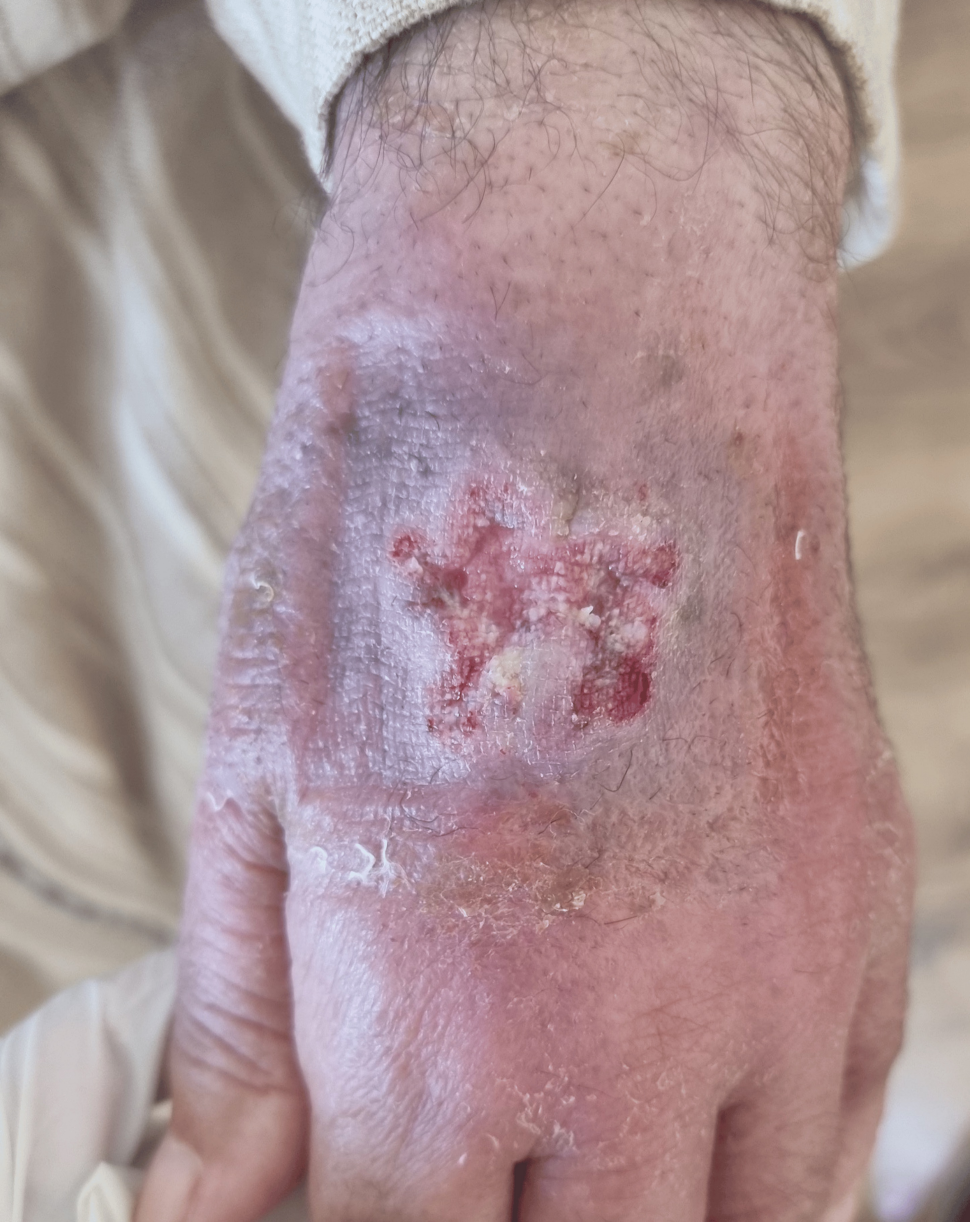
One week after completion of an eight-week course of intramuscular meglumine antimoniate, the lesion shows partial re-epithelialization, with decreased erythema and reduced crusting, though the ulcer remains incompletely healed.

The patient received systemic meglumine antimoniate therapy, which resulted in some clinical improvement (Figure 2).

**Figure 3:**
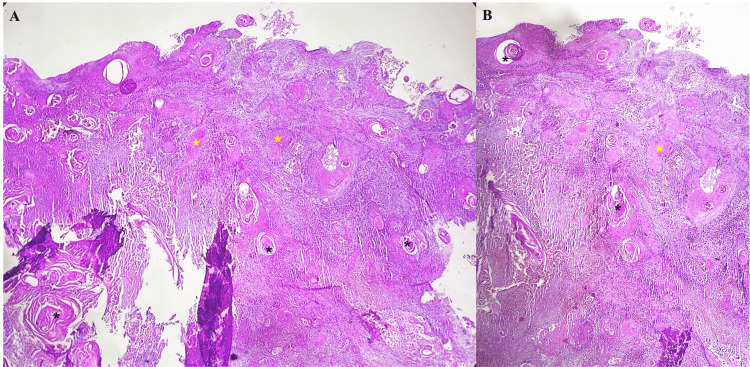
Histopathological features of cutaneous SCC: (A) A low-power view reveals broad infiltration of the dermis by irregular lobules and nests of atypical squamous cells (yellow asterisks), along with multiple keratin pearls (black asterisks) (Hematoxylin and eosin stain; original magnification ×40). (B) A higher magnification shows invasive nests of atypical keratinocytes with eosinophilic cytoplasm and prominent keratinization (keratin pearls), embedded in a desmoplastic stroma with a dense inflammatory infiltrate (Hematoxylin and eosin stain; original magnification ×100). SCC, squamous cell carcinoma

However, the chronicity of the lesion and its partial response to treatment raised concern for possible malignant transformation. A biopsy of the lesion was performed. Histopathological examination revealed a well-differentiated, infiltrative SCC (Figure 3).

**Figure 4:**
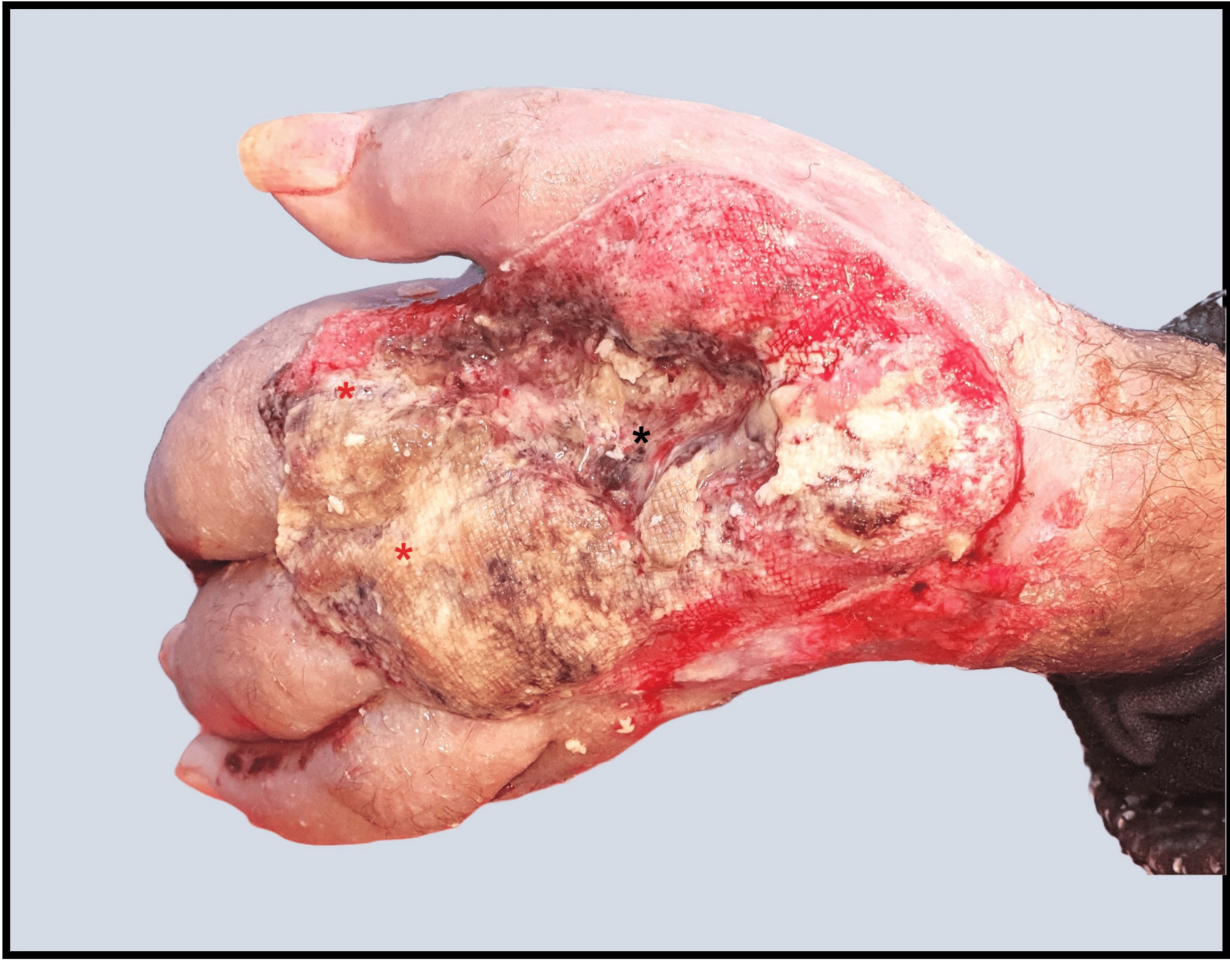
Progression of SCC two years after biopsy. A dorsal view of the left hand shows a large, ulcerated mass with nodular infiltration (red asterisk), involving the extensor musculature and underlying metacarpal bones (black asterisk). SCC, squamous cell carcinoma

**Figure 5:**
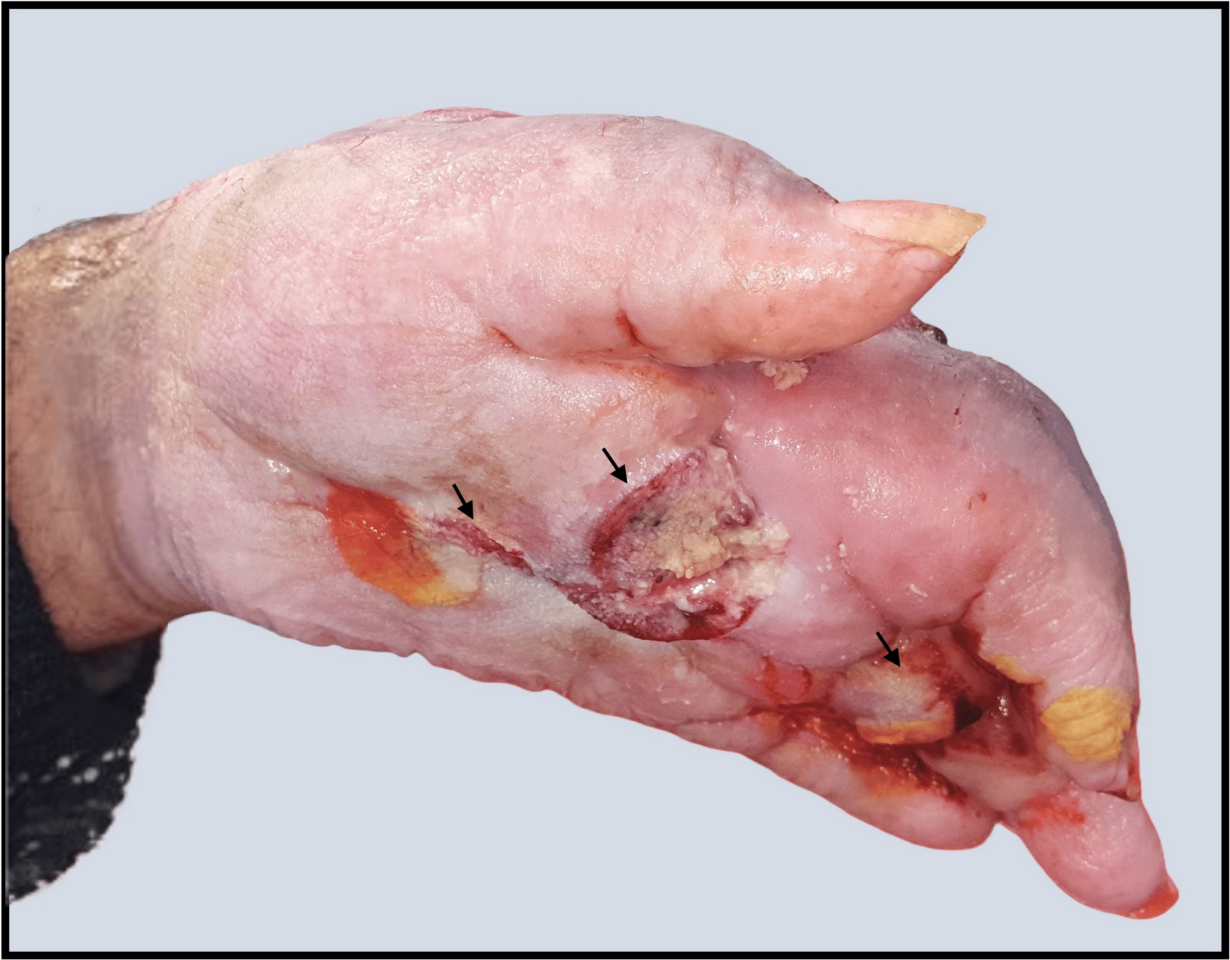
Palmar view of the left hand reveals full-thickness perforation of the skin with deep soft tissue destruction (black arrows), consistent with aggressive local invasion by the tumor.

Unfortunately, the patient was lost to follow-up for nearly a year due to cardiovascular health issues. Upon returning to our center, the tumor had significantly progressed, showing extensive local invasion and destruction of the underlying hand structures (Figure 4), including full-thickness perforation extending to the palmar region (Figure 5). Ultimately, the patient required amputation of the hand to control the advanced disease.

## Discussion

Leishmaniasis is a parasitic infection caused by flagellated protozoa of the genus Leishmania and is primarily transmitted through the bite of phlebotomine sandflies (i.e., Phlebotomus and Lutzomyia). The clinical manifestations of leishmaniasis are diverse, ranging from localized cutaneous lesions to severe systemic involvement [3]. Although diagnosing typical cases in endemic regions is generally straightforward, the disease’s broad spectrum of presentations can complicate the differential diagnosis. CL is often dubbed "the great imitator" because it may closely resemble several other dermatologic conditions and can even coexist with or unmask additional dermatoses [4].

Chronic inflammation has long been recognized as a critical factor in the malignant transformation of various skin conditions [1]. Prolonged immune activation, persistent oxidative stress, and continuous tissue damage create a microenvironment that favors carcinogenesis [5]. In chronic leishmaniasis lesions, the extended presence of Leishmania parasites, along with repetitive cycles of inflammation and tissue repair, can contribute to the development of SCC [6]. In this setting, inflammatory cytokines, particularly tumor necrosis factor-alpha (TNF-α) and interleukin-6 (IL-6), play crucial roles by promoting cell proliferation, inhibiting apoptosis, and encouraging angiogenesis, all of which are processes implicated in tumorigenesis [5,6].

The immune response to Leishmania is complex and involves both Th1 and Th2 pathways [7]. In cases of chronic infection, a shift in the immune balance may occur, resulting in immune dysregulation that favors malignant transformation [5,6]. Additionally, SCC developing in the context of chronic wounds, including scars from leishmaniasis, often employs immune escape mechanisms [8]. For example, the downregulation of major histocompatibility complex (MHC) class I molecules allows tumor cells to evade immune detection, while the expression of immunosuppressive factors such as PD-L1 contributes further to an environment that supports tumor progression [8].

Although several cases of leishmaniasis mimicking SCC have been documented, reports of SCC arising in leishmaniasis scars are less frequent. Even rarer are cases where active CL and SCC coexist at the same site. This association may result from the malignant transformation of a neglected chronic leishmaniasis lesion, but it could also represent the coincidental occurrence of SCC and a sandfly bite at the same location [9].

Given these complexities, it is essential to monitor chronic leishmaniasis lesions closely, especially those that persist beyond the standard treatment duration or display atypical clinical features. For high-risk patients, such as those with long-standing scars, chronic ulcers, or underlying immunosuppression, regular follow-up and prompt biopsy of suspicious changes are recommended to facilitate early detection and management of potential malignant transformation [1].

## Conclusions

This case illustrates a rare but serious complication of chronic CL: the development of SCC. It emphasizes the importance of clinical vigilance in monitoring non-healing or atypical leishmanial lesions, particularly in high-risk individuals. Early biopsy and histopathological evaluation are critical for timely diagnosis and appropriate management. In endemic regions, clinicians should remain alert to the potential for malignant transformation in chronic leishmaniasis, especially when standard treatment fails to achieve complete resolution.
